# Effect of diet on the evolution of gut commensal bacteria

**DOI:** 10.1080/19490976.2024.2369337

**Published:** 2024-06-21

**Authors:** Tanja Dapa, Karina B. Xavier

**Affiliations:** a Andalusian Center for Developmental Biology (CABD), Department of Molecular Biology and Biochemical Engineering, Pablo de Olavide University/CSIC/Junta de Andalucía, Seville, Spain; b Instituto Gulbenkian de Ciência, Oeiras, Portugal

**Keywords:** Gut microbiota, commensal bacteria, diet, nutritional regimens, evolutionary adaptation

## Abstract

The gut microbiota, comprising trillions of diverse microorganisms inhabiting the intestines of animals, forms a complex and indispensable ecosystem with profound implications for the host’s well-being. Its functions include contributing to developing the host’s immune response, aiding in nutrient digestion, synthesizing essential compounds, acting as a barrier against pathogen invasion, and influencing the development or regression of various pathologies. The dietary habits of the host directly impact this intricate community of gut microbes. Diet influences the composition and function of the gut microbiota through alterations in gene expression, enzymatic activity, and metabolome. While the impact of diet on gut ecology is well-established, the investigation into the relationship between dietary consumption and microbial genotypic diversity has been limited. This review provides an overview of the relationship between diet and gut microbiota, emphasizing the impact of host nutrition on both short- and long-term evolution in the mammalian gut. It is evident that the evolution of the gut microbiota occurs even on short timescales through the acquisition of novel mutations, within the gut bacteria of individual hosts. Consequently, we discuss the importance of considering alterations in bacterial genomic diversity when analyzing microbiota-dependent effects on host physiology. Future investigations into the various microbiota-related traits shall greatly benefit from a deeper understanding of commensal bacterial evolutionary adaptation.

## Introduction

The trillions of diverse bacteria that make up the human gut microbiota are crucial for the health of their host.^1,2^ Alterations in the gut microbiota that significantly affect its composition can affect host health and have been linked to many systemic and intestinal diseases, including inflammatory bowel disease, cardiovascular disease, diabetes, obesity, allergies, metabolic syndrome, and others.^2–8^ Switching from a diet low in fat and rich in fiber and plant polysaccharides to a diet high in saturated fat and simple carbohydrates is a common cause of gut microbiota alterations in urbanized societies consuming this type of diet. The effects of this switch are well understood at microbiota composition and metabolome levels.^9–13^ Still, it’s becoming abundantly clear that to fully comprehend the impact of the microbiota on the health of its host, we must go beyond our current understanding of species composition, identify the functional characteristics of the individuals who make up this community, and understand how they evolve across host generations and within the host lifetime.

**Figure 1.**
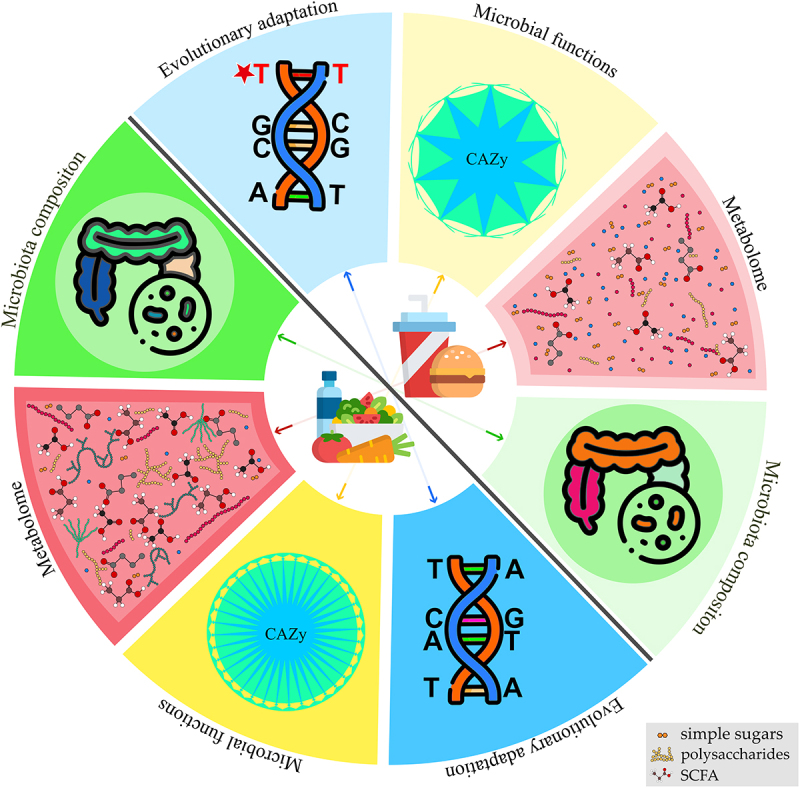
Effect of diet on gut microbiota and related functions. Diet influences various aspects of the gut microbiota. A diet rich in fiber and complex polysaccharides, as opposed to a diet rich in simple sugars and fat, promotes microbial diversity, thereby enhancing microbial functions. Alterations in gut metabolome are a crucial consequence of different dietary regimens. Diet affects gut metabolome directly by nutrient availability and, indirectly, as gut microbes process dietary nutrients into diverse metabolic products. Dietary fibers also influence the gene expression and production of gut microbiota enzymes. CAZymes (carbohydrate-active enzymes), involved in the degradation of dietary fibers, are produced by gut microbes, and their production decreases when the host consumes low-fiber diets. Short-chain fatty acids are important metabolic fermentation products resulting from the action of CAZymes on plant polysaccharides from dietary fibers. Thus, these metabolites decrease in abundance when complex polysaccharides are excluded from the diet. Furthermore, different diets imprint a genetic signature on the gut bacteria, due to adaptation to the available nutrients.

The gut microbiota comprises hundreds or thousands of different microorganisms, making it a tremendously complex habitat. The human intestine has 500–1000 different bacterial species, not counting other microorganisms.^14^ The biodiversity at the strain level may also be several orders of magnitude higher.^15,16^ Furthermore, the gut microbiota is constantly changing in response to environmental changes and alterations in host physiology (*e.g*., upon consumption of different diets and exposure to pharmacological treatments), adding to the functional variety of this ecosystem.^14,17–19^ Therefore, to understand the interactions between the microbiome and its host, it is crucial to study microbes at the inter- and intra-species levels. Here, we will discuss how nutrition influences the species diversity and evolution of gut commensal bacteria (Figure 1).

## The modern gut microbiota

Initial colonization of the human gut occurs during the first year of life^15,17–21^ when newborns are exposed to the maternal and environmental microbes.^22^ The gastrointestinal tract of newborns is colonized by between 10^13^ and 10^14^ microbes.^21^ Mode of delivery (vaginal or Caesarean section), early-stage feeding, as well as antibiotic treatment, were shown to determine gut microbiome signatures during the first year of life,^19,20,23–26^ and weaning from breastfeeding, rather than the introduction of solid food, led to the development of a mature microbiota.^20,27^ Twelve-month-old infants who continue to be breastfed while consuming solid food have a more immature microbiota composition than infants who cease receiving breast milk, and the latter have a faster transition to an adult-like composition.^20^ While the factors encountered during the early stages can have functional effects on the gut microbiota and, consequently, on its host later in its lifetime^28–30^ the composition of the colonic microbiota beyond the initial erratic stages is considered relatively stable within healthy adult individuals.^31–34^ In contrast, the microbiota of small intestine remains dynamic into adulthood due to dietary intake and subspecies variation.^32^

However, while unique and stable within an individual, the gut microbiota has substantially changed throughout human history.^35,36^ Along with changes in diet, other elements also altered over time, *e.g*., modernization of medicine, limited exposure to pathogenic microbes due to antibiotics, sanitation, Caesarean section delivery, and urban lifestyles. All these changes vastly improved human well-being in many aspects but also had negative repercussions on gut microbiota diversity,^9,19,24,35,37–43^ and while lower gut microbiota diversity does not automatically represent negative consequences for the host, as in the case of extinction of pathogenic parasites,^44^ it was shown that in general a higher microbial diversity implies a higher functional capacity of the microbial community, therefore potential health benefits for its host.^2,45–47^ Many studies show that the diversity in the gut microbiota seems to depend on nutrition, *e.g*., a diet rich in plant polysaccharides and fibers, rather than geographical location. The differences can be detected early in life, and persist through generations.^43,48,49^

Observations coming from human studies motivated a series of experiments performed on mice as a model organism. Namely, it was shown that a prolonged period of consumption of a diet poor in plant polysaccharides and fibers can irreversible extinct entire bacterial taxa.^50^ In a humanized mice model (*i.e.*, mice colonized with human microbes), when the mice were fed a diet poor in microbiota-accessible carbohydrates (diet lacking plant polysaccharides and fibers), the changes in microbiota composition were largely reversible upon diet change within a single generation, but irreversible when mice consumed diet low in polysaccharides over several generations.^50^ The majority of the irreversibly extinct taxa (77 out of the 114) belonged to the order Bacteroidales.^50^ Only the administration of the lost taxa, in combination with the consumption of a diet rich in microbiota-accessible carbohydrates, restored the initial microbiota.^50^

While the reduction of bacterial diversity is associated with a decrease in the representation of genes encoding carbohydrate-active enzymes, and, consequently an indirect loss of functions provided to the host by its microbiota, changes in the host diet can also cause changes in the behavior of certain bacteria (*i.e*., through changes in gene expression) that can also cause a direct impact on the wellbeing of its host. One example is the case of the bacterium *Bacteroides thetaiotaomicron*, which can switch from consuming complex carbohydrates present in dietary fibers to start consuming glycans present in the host intestinal mucus layer when the polysaccharides derived from plant and fibers are absent from the diet.^51–53^ One of the landmark studies addressing the mechanisms behind microbiota-related disorders showed that the lack of dietary fibers promotes the expansion and activity of colonic mucus-degrading bacteria.^54^ This resulted in thinning of the mucus layer, which facilitated access to epithelial and, consequently, lethal colitis caused by a murine mucosal pathogen, *Citrobacter rodentium* .^54^ Diet also had severe consequences during *Clostridioides difficile* infections, as mice consuming a diet lacking dietary plant polysaccharides had a significantly worse course of the infection.^55^ The addition of dietary polysaccharides to a diet resulted in the outgrowth of microbiota members, associated with the production of short-chain fatty acids, *i.e*., products from degradation of complex polysaccharides – acetate, propionate, and butyrate, which consequently decreased *C. difficile* fitness.^55^ Additionally, a shift from a mouse regular diet to a diet with reduced fibers (high-fat diet) resulted in a boost of *Salmonella* Typhimurium gut colonization, promoted by fat-elicited bile salts.^56^

Beyond the observation that the host diet correlates with the changes in microbiota composition with consequences for susceptibility to infections by intestinal pathogens, other observational studies have also revealed associations between microbiota alterations and different common metabolic disorders, *e.g*., obesity, malnutrition, type 1 and 2 diabetes, cardio-metabolic and metabolic dysfunction-associated fatty liver disease, and these descriptive studies are now moving toward understanding the mechanism behind these observations.^2,57^ After the initial discovery, where microbiota from an obese mouse was transplanted into a lean mouse, which caused increase in weight gain in lean mice,^58^ an elegant way to study this phenomenon emerged.^59,60^

Many reports followed, providing evidence that diet can regulate microbiota-linked disease, raising the possibility that a controlled microbiota transplant, coupled with a regulated diet, could treat or prevent these diseases.^61,62^ However, consuming a diet that promotes gut microbiota diversity alone may not be sufficient to prevent microbiota-associated disorders. Indeed, a recent study in which 21 healthy volunteers were provided with a homogenized diet in a uniform chopped salad format for a period of 7 days showed that consuming a homogenized diet alone does not reduce inter-individual variation in microbiome composition, metabolic output, and microbiome-dependent metabolites.^63^ In fact, in this study, host identity and age, rather than diet, were shown to be the main contributors to the microbiome-dependent metabolite variability.^63^ To reverse microbiota-linked diseases caused by diet alterations, fecal microbiota transplantation or other ways of altering the microbiota may be required. New studies are now focusing on coupling diet together with targeted microbiota transplantation.^62^ Targeted and personalized transplantation of gut microbiota members, coupled with a microbiota-stimulating diet, may prevent many disease states (current knowledge is reviewed in Ref).^4^

## One bacterial species, many phenotypes – emergence of novel strains within individuals

Although the microbiota in adult individuals becomes more stable, compared to the erratic early stages observed in early life, diet, host-extrinsic factors, and many other environmental can affect the composition and functions of the gut microbiota.^32,33,64^ Additionally, in terms of species composition, the apparent microbiota stability might be hiding intra-species variations that can be important to maintain the diversity and functional robustness in response to constant perturbations. In a study where microbiota real-time samples coming from ileal stoma were analyzed, it was shown that even when absolute microbiota biomass changes with the feed cycle (absolute numbers of microbes in the distal small intestinal increased 10-fold only 2 h after the breakfast), the composition at the taxon level remained stable.^32^ However, deep metagenomic sequencing showed that this apparent taxon stability hides very dynamic alternations of bacterial strains within individual taxa, which correlated with the feeding cycle.^32^ While the strain differences were bigger between individuals than within an individual, many taxa within each individual were also constituted by multiple different strains, which oscillated in relative abundance over the feeding cycle.^32^ Change in abundance of different strains within hours demonstrates how plastic the gut microbiota really is, which could be crucial for maintaining the diversity within the host as different co-existing strains could benefit from, and bloom in response to, different conditions.

The appearance of new strains was also reported in a controlled experiment, within the mice gut. A colony of germ-free mice was colonized with a defined community of 12 fully sequenced phylogenetically diverse representative taxa of murine gut microbiota and was followed for 6 years.^65^ While the gut of mice remained stably colonized with all 12 members (over several generations of mice), new strains emerged within individual taxa, showing different gene expression while consuming different nutrients, and the proportions of different strains rapidly changed upon a dietary shift.^65^

All this shows that microbial species colonizing the mammalian gut are composed of many different strains. Strains of each species differ substantially between them. Intra-species variation may reflect different functional capabilities, as a result of differences in gene sequences or even the number of copies of each gene they carry.^66^

The host diet is one of the elements that could significantly add to intra-species variation by influencing bacteria at the strain level, as was shown for the bacterium *Prevotella copri*. While the role of *P. copri* is not clearly understood in the balance between health and disease of the host,^67,68^ it is clear that this bacterium is associated with the consumption of a diet high in plant polysaccharides and fibers (an agrarian-type diet – fruit and vegetables).^34,69^ In human gut microbiota the high level of intra-species genetic diversity of *P. copri*, which is higher in populations with non-urban lifestyles, seems to be a consequence of exposure to different diets.^70^ Analysis of ancient stool samples suggested that the underrepresentation of *P. copri* in populations with urban lifestyles was led by urbanization itself, indicating the role of diet in this process.^70^ Additionally, sequences of the gut metagenome of healthy Italian adults with different dietary habits (*i.e*., omnivores, vegetarians, and vegans) separated *P. copri* at the strain-level based on the diet. While the abundance of specific strains of *P. copri* was not significantly associated with any diet type, analysis of pangenome clearly separated the three different nutritional groups, and genes involved in the degradation of complex polysaccharides were associated with a vegan diet.^71^

Further proof that the diet affects *P. copri* at the strain level comes from a study with a whole genome sequence of 83 *P. copri* isolates from the stool of 11 healthy individuals. The genome sequences of these isolates revealed an extensive genomic diversity within and between different hosts, and many of the host-specific genes were in a *susC*-like gene, a gene responsible for polysaccharide transport.^72^ This genomic variation was further linked to functional variation, as isolates grew differently on predicted plant polysaccharides.^72^ Within-species genomic variation, which was mainly in genes responsible for the degradation of different plant polysaccharides, could result from several selective pressures from the environment, *e.g*., different nutritional diets, different host genetic backgrounds, different health status, and drug consumption. However, the fact that multiple strains of *P. copri* came from the same host indicates that different isolates colonize different regions of the intestine, or, alternatively, indicates a possible polysaccharide cross-feeding, where different isolates work together by complementing one another to grow in the presence of more polysaccharides. To support the latter hypothesis is the fact that the population of *P. copri* coming from a host with multiple *P. copri* isolates was able to catabolize a greater diversity of polysaccharides than any individual strain alone.^72^

All these data highlight the need to consider the emergence of different strains as a result of evolution when interpreting microbiota-related phenotypes.

## Within-host evolution

Several hypotheses that the host and its microbiota evolved as a single evolutionary or organizational unit resulted from the strong connections between the two, *i.e*., hologenome or holobiont hypothesis.^73,^^74–79^ However, while this may be a plausible hypothesis for vertically transmitted symbioses, *e.g*., intracellular bacteria of insects, is much less likely that systems like the human microbiome evolved as a unit with its host.^80,81^ Strong evolutionary conflicts and/or cooperation between the host-microbiota, and within the microbiota itself, are main concerns acting against evolution as a single unit.^82–86^ Even though this knowledge is essential for understanding how the gut microbiota and the host interact, we still know very little about how the gut microbiota evolved properties that benefit the host and how the host immune system evolved to allow the presence of trillions of different microorganisms.

While the coevolution between the host and its microbiota remains poorly understood, new knowledge is emerging on the within-host evolution of the gut microbiome.^87^ Since the gut microbiota potentially inhabited the host intestine for millions of years,^88–92^ it could be expected that in a relatively stable environment, all evolutionary beneficial adaptations would have occurred long ago and that current optimal host-microbiota connection foreclosed any adaptive within-host evolution, allowing only neutral or very weakly beneficial mutations.^93,94^ However, it has recently become clear that gut microbiota can still evolve within its host, and on a very short timescale. The gut environment is far from unchanging; diet, geography, drug treatments, and others factors that put the bacteria within the gut microbiota under constantly changing selective pressures strongly influence the microbial communities, including at the single species gene level. Studies of microbiota evolution within human individuals showed bacteria can be under long-term purifying selection,^95,96^ neutral selection,^97,98^ but also under adaptive selection.^99^ It is important to note that the study showing neutral evolution within the human gut^98^ resulted from following the evolution of an *Escherichia coli* isolate, which is present in the human gut at relatively low frequencies (~10^8^ colony-forming units, CFU), but adaptive evolution was shown for a highly abundant gut microbiota member, *Bacteroides fragilis*^99^ (>10^11^ CFU). The difference observed might be explained by the differences in effective population sizes which have an impact on the patterns of evolution.^100,101^ Zhao et al.^99^ explored the adaptive evolution of *B. fragilis* isolates in healthy humans by combining culture-based population genomics and metagenomics. They identify *de novo* mutations, which emerged in 12 healthy individual human hosts. Independent emergence of similar mutations, *i.e*., parallel evolution, was observed in 16 genes of *B. fragilis*, many of which were related to cell-envelope biosynthesis and polysaccharide utilization. Furthermore, the addition of public metagenomic data revealed that a common adaptive mutation of *B. fragilis* occurs frequently in the gut microbiome of North Americans and Europeans individuals (but not Chinese individuals), suggesting that regional or dietary factors play a role in driving the evolution.^99^ Moreover, a model-based framework that assessed the evolutionary dynamics within and between hosts by employing a panel of metagenomic samples further demonstrated that the gut bacteria can evolve on human-relevant timescales.^102^ This study emphasized the connections between within-host short-term evolutionary dynamics and long-term evolution across hosts. Throughout the course of six months, genetic alterations might occur within the same host, and these changes are almost always the result of evolutionary changes rather than replacement by closely related strains. However, comparisons of data received from adult twins showed that replacement can eventually exceed local adaptation.^102^

**Figure 2.**
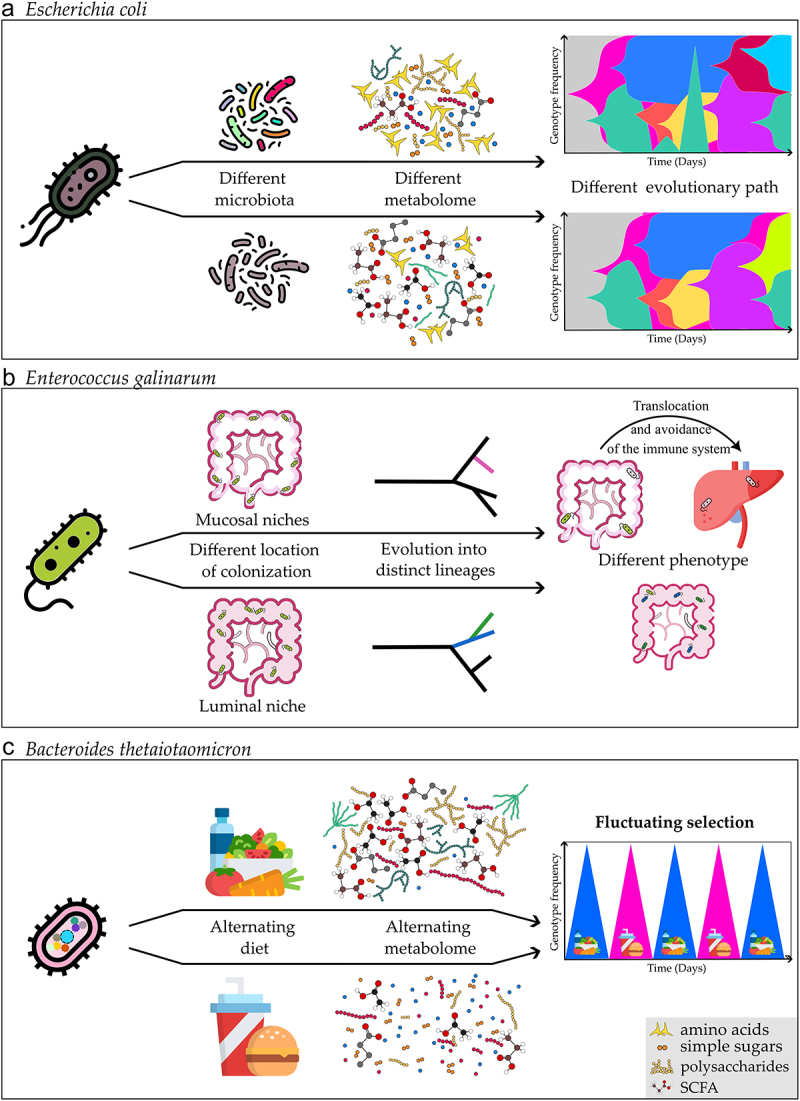
Evolutionary adaptation at the single species level. Different factors affect the evolutionary adaptation of a single bacterium. **a)** the presence, absence, or differences in composition of other microbiota members (*i.e*., in *E.* *coli* mono-colonized animals versus in the presence of other microbiota members) causes changes in the metabolome, and microbe-microbe interactions which impose different pressures selecting for different evolutionary paths.^106^ Muller plots (right illustrations on the top panel) represent the emergence of a beneficial mutation that changes in frequency through time (each color corresponds to a single mutation). **b)** Bacteria colonizing different regions of the gut can select for the evolution into distinct lineages^119^ in autoimmune-prone mice *Enterococcus gallinarum* adapted to different phenotypic lineages while adapting to colonize either luminal or mucosal intestinal niches. Mucosal adapted lineages evolved to better avoid the immune system facilitating bacterial translocation to the liver. **c)** Different nutritional regimens of the host result in a different gut metabolome, and weekly shifts between different diets result in periodic selection in *B.* *thetaiotaomicron*, during which different beneficial mutations peak.^105^.

It is particularly challenging to interpret the intraspecies evolutionary dynamics in humans given the complexity of the gut microbiota and the difficulties associated with transmission between hosts, even among adults. Because of this, exploring intraspecies genetic diversity and the environmental variables affecting the occurrence of *de novo* mutations in controlled and simplified models with experimental evolution can be a valuable alternative. Such approaches have enabled us to identify some of the causes, and pace of the commensal bacteria’s evolutionary adaptability in the mammalian intestine using mice as model organisms. It has been shown through studies investigating the evolution of a single gut species that adaptive evolution can occur as rapidly as within a few weeks or months when adapting to the mouse gut changing environment.^103–105,106^ Interspecies competition, *i.e*., microbiota composition, was shown to be one of the major ecological components that directly influences evolution (Figure 2a). *E. coli* evolution is more predictable across hosts in germ-free animals where the gut microbiota is lacking, and the evolutionary path can be altered by including just one extra gut microbiota member, *Blautia coccoides*^106^ because the presence of a competitor species was sufficient to alter the gut metabolome.^106,107^ Most studies showed that the gut bacteria adapted mostly by changing metabolic capabilities.^106–110^ Other factors, such as the animal’s age^111^ or immune status,^112^ also influence the evolution of *E. coli* in the mouse gut, with older animals evolving bacteria that are better at tolerating stressful environments, which was correlated with the older mice’s more inflamed gut, as opposed to younger mice that evolve toward metabolic adaptation.^103,111^ Furthermore, the changing selective pressures in immune-compromised mice do not automatically increase the speed of evolution, as it was demonstrated that the absence of an adaptive immune system (Rag2^−/−^ knockout mice, *i.e*., mice lacking lymphocytes) slows the evolution of *E. coli*, even though this was probably caused by the differences in the microbiota composition of the immunocompromised animals.^113^

In the mammal gut, interactions between different species and kingdoms also influence the rate and mode of evolution.^114,115^ If there are no existing *E. coli* strains in the gut community, an invader *E. coli* rapidly evolves by *de novo* mutations; however, if the gut is already colonized by an *E. coli* strain, the course of evolution is altered. In that case, horizontal gene transfer (HGT) can drive the rapid evolution of the invasive *E. coli* strain. The rapid phage infection of the nonresident invader *E. coli* was caused by two bacteriophages carried by the resident strain.^114^ Evidence of the importance of HGT in eco-evolutionary processes was also found in the human gut microbiota.^102,115,116–118^

Importantly, recent research has demonstrated that the evolutionary path of gut bacteria is influenced by the environmental niche in which they evolve.^119^ *Enterococcus gallinarum*, a model gut pathobiont, has evolved into two distinct lineages within the gut of autoimmune-prone mice^119^ (Figure 2b). One lineage specialized to colonize the luminal niche, and the second the mucosal niche of the mice gut.^119^ The lineage, which evolved to better colonize the mucosal niche of the intestine, improved the ability to avoid the immune system detection and clearance.^119^ This lineage has also shown an increased ability to translocate and survive within the mesenteric lymph nodes and liver, increasing intestinal and hepatic inflammation.^119^ This phenomenon was not exclusive to a single bacterium but was also observed in *Lactobacillus reuteri.*^119^ This landmark study demonstrated how short-term evolution of specific species within the host can have microbiota-mediated effects on physiology with consequences for host disease.

## Bacterial evolution in response to the host diet over a long evolutionary timescale

**Figure 3.**
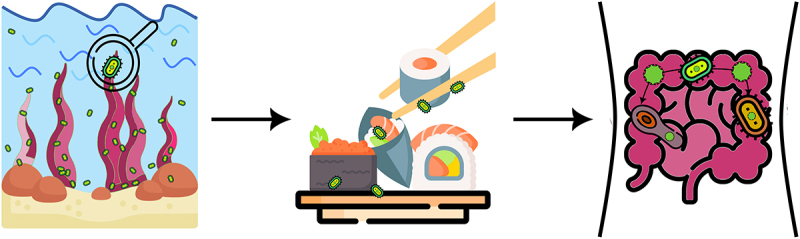
New food – old solution: Acquisition of novel functions through lateral gene transfer. Commensal gut bacteria, lacking enzymes necessary for the degradation of specific compounds introduced in the diet, can acquire genes encoding these enzymes by lateral gene transfer. This was shown in the case of the bacterium *Bacteroides plebeius* .^127^ Glycoside hydrolase, which cleaves porphyrin, a polysaccharide abundant in the red algae *Porphyra* sp., was acquired by *B.* *plebeius* through lateral gene transfer from the marine bacterium *Zobellia galactanivorans* .^127^ Evolved strains of *B.* *plebeius*, with this acquired enzymatic capabilities, were detected in the Japanese population. This population is known for consuming a diet rich in porphyran found in algae commonly used to wrap sushi.^127^ Detection of these evolved *B.* *plebeius* strains in the Japanese population are a compelling example of how gut bacteria can adapt to novel dietary components through lateral gene transfer.

Among all types of nutrients, glycans are the major source of energy storage and the planet’s primary source of biomass.^120^ According to theoretical models in natural products, the potential wide range of isomers for all glycan structures is > 10^12^ (hexasaccharides),^121^ despite that, the human body only has 17 different carbohydrate-active enzymes (CAZymes) with which it can break down glycans.^122,123^ In contrast, gut microbiota members encode thousands of different CAZymes and associated proteins, *e.g*., transport systems, uptake of polysaccharides, depolymerization, which ferment glycans into host-absorbable short-chain fatty acids (SCFAs).^123,124^ Consequently, it should come as no surprise that among the many advantages the gut microbiota offers its host, one of the most fundamental is the breakdown of nutrients and supply of the host body with energy from dietary polysaccharides. The bacterial members of the phylum Bacteroidota (formerly known as Bacteroidetes) alone can degrade a myriad of different polysaccharides as they possess a vast number of diverse CAZymes,^121^ an average of 137 per genome,^123^ with individual species having between 100 to 300 different enzymes.^125^ Most known CAZymes found in gut microbiota members target polysaccharides from terrestrial plants.^126^ However, throughout the evolution of humans’ gut microbiota demonstrated a high degree of plasticity to adapt to novel nutrients. The breakdown of seaweed’s polysaccharides is a famous example.^124,127^ Commensal gut bacterium *Bacteroides plebeius*, found in the feces of healthy Japanese individuals, was the first gut bacterium in which a glycoside hydrolase responsible for cleaving a polysaccharide known as porphyrin (abundant in red algae of the *Porphyra* species) was found^127^ (Figure 3). This groundbreaking research showed that glycoside hydrolase originated in the marine bacterium *Zobellia galactanivorans* and that *B. plebeius* laterally acquired this gene from marine bacteria.^127^ Long evolutionary timescales allowed the gut bacterium to acquire genes that enable it to digest polysaccharides solely found in the Japanese diet, *i.e*., porphyrin is rich in nori made from *Porphyra*, which is used to wrap sushi.^127^ More recent studies further showed that members of *Bacteroides*, the most predominant genus of Bacteroidota in the human intestine, also possess genes that degrade agarose,^128^ alginate,^129,130^ and laminarin.^131^ Although marine Bacteroidota are physiologically distinct from intestinal *Bacteroides*, they share mechanisms for the degradation of these polysaccharides, as the genes implicated in the processes have close homologs in marine Bacteroidota.^132^ Recent research has revealed that acquiring genes for seaweed polysaccharide digestion from marine environments to gut microbiota is more widespread than previously thought.^133^ DNA mobilization occurred by a number of distinct events, *e.g*., two known for porphyran,^127,134^ and included in several bacterial genera, including in members of gut-resident phylum Bacillota (formerly known as Firmicutes).^133^

Changes in the host diet can also select for mutants in intestinal pathogens with consequences for infection outcomes. Trehalose, a non-reducing disaccharide of glucose, was extensively introduced into human diets by the food industry as a preservative in the early 2000s in Europe and North America. Collins and colleagues demonstrated that major epidemic and hypervirulent strains of *Clostridioides difficile* have mutations that enable them to better metabolize trehalose than other *C. difficile* strains.^135,136^ They further showed that this metabolic property confers a competitive advantage to these strains in the presence of complex intestinal microbiota communities. Their studies provide strong support for the proposal that the introduction of trehalose into the diets of European and North American populations was an important factor in promoting the emergence and spread of epidemic *C. difficile* ribotypes that are more efficient in metabolizing trehalose. These strains have become a leading cause of hospital infections in Europe and North America.^135,136^

## Bacterial evolution in response to the host diet over a short evolutionary timescale

As remarkable as the gut microbiota’s plasticity over long evolutionary timescales is, as discussed above, bacteria in the host’s intestine can also evolve at much faster rates. Nutrition as a short-term external environmental factor also impacts the evolution of gut bacteria. Indeed, in our previous study we showed that one of the predominant members of the human gut microbiota, *Bacteroides thetaiotaomicron*, can adapt quickly to the mouse gut by acquisition of novel mutations, in a period as short as three months, and different dietary regimens of the host can leave a genetic signature in this bacterium.^105^

In addition to enhancing its ability to colonize new environments (a human isolate was studied in the mouse gut), the bacterium evolved to respond differentially to the various diets that the mice consumed throughout the evolutionary experiment (*i*, high fiber – high plant polysaccharide diet (Standard Diet – SD); *ii*, high fat – high simple sugar diet (often called Western Diet – WD, to refer to diets of European, North American and other populations with urban life-styles, which consume processed food rich in these compounds); *iii*, weekly alternation between the two diets (Alternation Diet – AD)).^105^ Interestingly, the bacterium had greater genetic diversity after weekly changes between the two diets, suggesting that nutrient switching causes a selective pressure that increases polymorphism.^105^ Additionally, some of the mutations selected during the AD regimen were not found in the animals that were on the constant SD or WD, therefore the AD left a unique genetic signature. Moreover, our study showed that since there was a greater correlation between mutations and the gut metabolite environment than between mutations and microbiota composition, the former, rather than the latter, had a more substantial influence on bacterial evolution.^105^

The results from this study also enable us to propose that genetic diversity can be used as a biomarker of dietary differences between individuals, as analyses on the expected power of within-species mutational profiles to predict the various dietary regimens revealed that within-species mutations are at least on par, if not better, than the microbiota composition and the metabolome,^105^ which are frequently mined for their potential as biomarkers.^1,137^ Integration of all three datasets: mutational profile, metabolome, and microbiota, revealed that only the mutation dataset can distinguish samples from all three different dietary regimens. The cross-validation error rates showed that the mutation dataset is the only dataset that exhibits a misclassification of less than 20% for all dietary regimens. Metabolites were very efficient at identifying samples from groups on a WD regimen but had an error rate close to 40% at identifying other regimens.^105^ Metabolites change very quickly with changes in diet, leaving no “memory” effect, and therefore cannot be used to differentiate constant regimens from alternation diet regimens. However, as the three different regimens selected for unique mutations in all three regimens, mutations can be a better biomarker, as it leaves a signature even in the AD regimen.

*B. thetaiotaomicron*, although preferably consumes complex carbohydrates from dietary fibers, can also consume host-derived glycans, a phenotype of some gut bacteria that enables them to survive in the intestine even when plant polysaccharides and fibers are absent from the diet.^51–54^ We observed that the consumption of a high-fat/high-simple sugar diet selected for mutations in *B. thetaiotaomicron* with increased growth capacity in host-derived glycans from mucin.^105^ Moreover, these mutations fluctuated in the AD regimen in response to the weekly alterations between the two diets, thus showing genetic and phenotypic fluctuations in response to diet (Figure 2c). This selection for mutants better at consuming host glycans from mucin in WD could be involved and responsible for the observation described previously by others, that animals fed diets low in fibers have thinner mucus layers.^54^

Considering this new information, earlier findings should be reevaluated. While it is undeniable that a shift in diet causes changes in microbiota composition and the metabolic environment, our studies suggest that evolutionary changes in these microbes may also enhance some microbiota-dependent phenotypes affecting host physiology. One such phenotype is the thinning of the mucus layer observed in animals consuming low-fiber diets. Previous studies have linked this phenomenon to a shift in the microbiota’s composition, characterized by an increased relative abundance of recognized consumers of host-derived glycans, such as *Akkermansia muciniphila* and *Bacteroides caccae*.^54^ Considering our findings, we advocate for investigating the role of selection for novel mutations within members of the microbiota, particularly in those members that increase in abundance in response to the perturbations under evaluation, to understand the mechanisms behind the causal effects of the microbiota.

## Conclusions and future perspectives

The importance of the host`s nutritional diet on the gut microbiota composition is undeniable. It has a direct impact on microbial composition, gene expression, and activity, consequently influencing the gut metabolome and the functions that the microbiota provides to its host. While the effect of dietary intake on microbial genotypic diversity has largely been ignored, recent studies now provide evidence for the impact of nutrition on both short- and long-term evolution in the mammalian gut. This evidence ranges from experimental evolution in murine hosts to long-term monitoring of human cohorts, highlighting nutrition as a crucial mechanism shaping microbiota functions. Future studies, which must consider a broader spectrum of gut microbiota members, including those colonizing different gut segments, glycan consumers, polysaccharide degraders, vitamin producers, various short-chain fatty acid (SCFA) producers, pathobionts, *etc*., will unveil more about the relevance of bacterial evolutionary adaptation.

In this review we focused on the impact of fiber and complex polysaccharide degradation since a large body of research has demonstrated its significance for the microbiota. However, recently is becoming increasingly clear that also other macronutrients, *e.g*., dietary proteins, largely affect intestinal microbiota, from the compositional and functional view.^138,139^ While further work is needed on this less explored topic, current knowledge already provides evidence to support the importance of dietary proteins on the microbiota and on the health of the host. In certain cases, when there is an excess of proteins in the intestine and those don’t get absorbed by the host, the microbes can ferment the remaining proteins in the colon, resulting in an increase of host-damaging protein derivatives, *e.g*. branched-chain amino acids and nitrogen.^139^ Additionally, it is very likely that dietary proteins and other macronutrients are also a source of selection within host evolution. It will be interesting to understand if dietary proteins, namely in the absence of fibers from the diet, will drive bacterial evolution against or toward benefiting host’s health.

This knowledge, on how gut microbes can evolve within the host in response to perturbations, related to dietary fibers or other macronutrients, will help us determine how to leverage it to our advantage for maintaining the stability and diversity of the gut microbiota.
